# Tritium-Labeled Compounds II. General-Purpose Apparatus, and Procedures for the Preparation, Analysis, and Use of Tritium Oxide and Tritium-Labeled Lithium Borohydride^1^

**DOI:** 10.6028/jres.063A.012

**Published:** 1959-10-01

**Authors:** Horace S. Isbell, Joseph D. Moyer

## Abstract

A general-purpose manifold is described, which is useful for numerous procedures involving tritium gas and tritium-labeled materials.

Methods are given in detail for (a) converting tritium gas to tritium oxide, (b) preparing tritium-labeled lithium borohydride, and (c) conducting a variety of reactions in a closed system. Auxiliary equipment is shown, including water traps, reaction flasks, and apparatus for preparing solutions and making filtrations in closed systems. Methods are presented for assaying tritium-labeled water by (a) dissolving it in a phosphoric anhydride-sulfuric acid solution and counting in a windowless, gas-flow, proportional counter, or (b) converting it to hydrogen-*t* and assaying it in an ionization chamber. The use of tritium-labeled lithium borohydride is illustrated by the preparation of d-galactitol-*1*-*t* from d-galactose. Subsequent papers will describe the use of this apparatus in the synthesis of tritium-labeled carbohydrates. The detailed description of techniques and apparatus should be helpful to others who are interested in using tritium.

## 1. Introduction

In a program sponsored by the Division of Research of the Atomic Energy Commission at the National Bureau of Standards, efficient methods have been developed for the production of C^14^-labeled carbohydrates [1 to 16].^2^ Attention has now been directed to the development of methods for the synthesis, analysis, and study of tritium-labeled materials. As the first step in the program, a convenient method was developed for the assay of nonvolatile, water-soluble tritium compounds [17]. Undoubtedly, this and other improved methods of analysis [18 to 21] will lead to rapid expansion in the use of tritium compounds for research.^3^

The production of tritium-labeled carbohydrates required the development of apparatus and techniques for conducting a variety of operations in closed systems. These include: (a) Dilution of tritium with hydrogen, (b) removal of samples of gas for radioassay, (c) conversion of tritium to tritium oxide, (d) purification of materials by filtration and crystallization in closed systems, (e) exchange of tritium with hydrogen in solids, (f) recovery of hydrogen-*t* released in chemical reactions, and (g) circulation of hydrogen-*t*, in catalytic reductions. The following sections describe convenient equipment for conducting these and other procedures.

Detailed directions are given for the preparation of tritium-labeled lithium borohydride. This versatile reagent makes possible a wide variety of reactions for introducing tritium into specific positions in organic compounds. Use of tritium-labeled lithium borohydride is illustrated by the preparation of d-galactitol-*1*-*t*^4^ from d-galactose. Other applications of the reagent will be described in future publications.

A convenient procedure is described for the radioassay of water-*t* in a proportional counter. The water-*t* is dissolved in a phosphoric anhydride-sulfuric acid solution and counted in a windowless, gas-flow, proportional counter in a manner similar to that used for assaying C^14^-labeled compounds in solution [23].

## 2. Apparatus and Procedures

### 2.1. General-Purpose Manifold^5^

**Figure 1:**
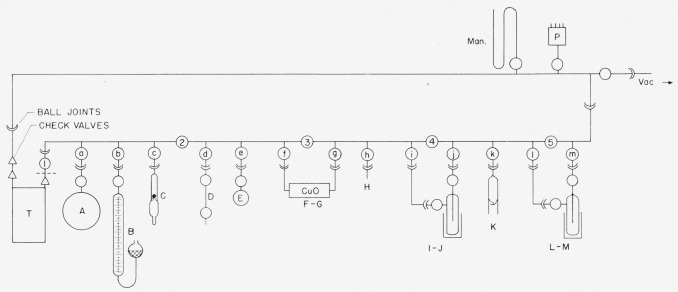
General-purpose manifold. ---- Sintered-glass filter. *T* Automatic Toepler pump. *P* Pirani vacuum gauge. Man. Mercury manometer. *A* Hydrogen-*t* storage flask. *B* Gas buret. *C* Tritium in break-seal tube. *D* Gas-sampling tube. (See fig. 3). *E* Water-storage flask. *F–G* Copper oxide furnace. *I–J* and *L*–*M* Cold traps. (See fig. 2). *K* Break-seal receiving tube.

**Figure 2:**
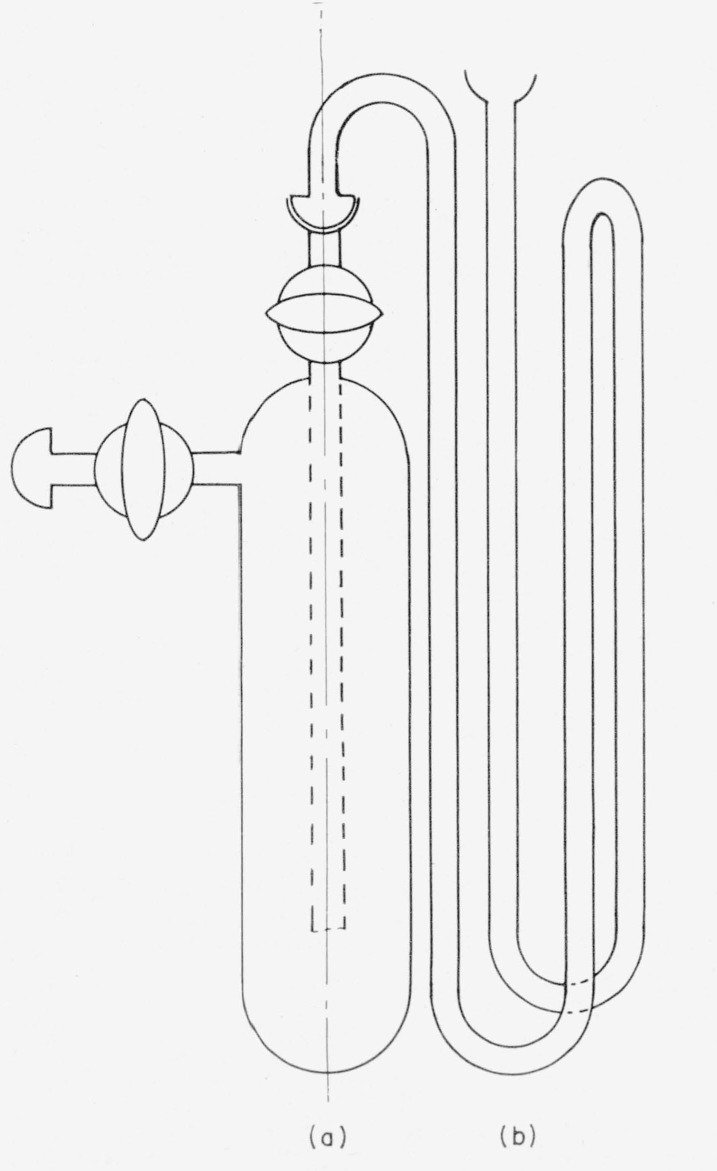
Interchangeable traps.

The general-purpose manifold shown in figure 1 is constructed from high-vacuum-grade ball-joints, stopcocks, and other parts. In figure 1, the stopcocks in the primary line are numbered; those connecting to auxiliary equipment are lettered. The commercial, automatic Toepler pump, *T*, is operated by an auxiliary pump activated by a lock-up or latch relay. A fritted-glass filter, just above the check valve on the pressure side of the Toepler pump, prevents entrainment of particles of mercury. To conserve space, the copper oxide furnace (for converting hydrogen-*t* to water-*t*) is placed above and behind the main line; it is kept in place, but is bypassed when not in use. Interchangeable, low-temperature traps (fig. 2) are used for collecting and storing water and other volatile liquids. Part (a) of this trap is ordinarily used alone, but for more efficient removal of condensable gases, a multiple trap (b) is included [26]. The two components are immersed in the same freezing bath, and can be used in combination (on the same outlets as a single trap). The traps are shown symbolically at (*I*–*J*) and (*L*–*M*) in figure 1. The gas buret, *B*, is used not only for measuring and dispensing hydrogen, but also for transferring gas to the reaction flasks. At the close of each preparation, hydrogen-*t* in the system is either converted to water-*t* in the copper oxide furnace (*F–G*), or is pumped into flask *A* attached to the manifold; any residual gas is stored in the Toepler pump. The arrangement given in figure 1 is used for the production of tritiated water. Other arrangements are used for special purposes; these will be described in subsequent reports on specific products.

### 2.2. Dilution of Tritium with Hydrogen

The apparatus of figure 1 is assembled, made vacuum-tight,^6^ and evacuated. The system is then filled with dry hydrogen, introduced at *H*, and the quantity of hydrogen required for the desired dilution is drawn into gas buret *B.* The stopcock to *B* is closed and the system is evacuated to a pressure of less than 20 microns of mercury. With all stopcocks closed, the “break-seal” joint connecting the tritium sample, *C*, is ruptured by means of a steel ball operated by a magnet. Then, with stopcocks 1, 2, and *a* closed, the tritium and the hydrogen are mixed in *B* by opening stopcocks *b* and *c* and raising and lowering the leveling bulb containing mercury. Finally, the hydrogen-*t* is transferred to the evacuated reservoir *A* for storage, leaving any desired amount in *B.* Residual hydrogen-*t* in the line is drawn into the Toepler pump and held for future use. The empty tube, *C*, is removed and can be replaced by other apparatus.

### 2.3. Radioactivity Assay of Hydrogen-*t*

**Figure 3:**
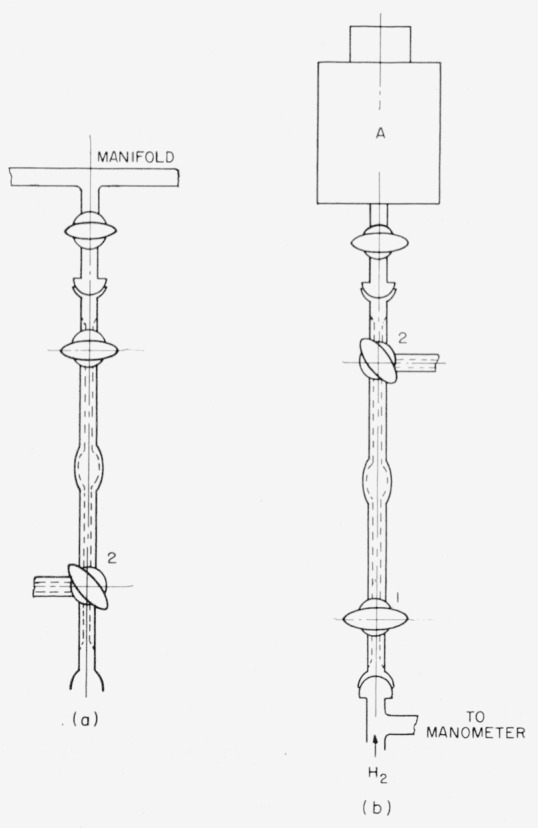
(a) Gas sampling tube and (b) Tube connected for transfer of sample to ionization chamber.

The gas in the manifold can be sampled at any desired time by means of the gas-sampling tube shown in figure 3(a). The tube is attached to the manifold at any available outlet. With the stopcock to the manifold closed and stopcock 1 of figure 3(a) open, the gas-sampling tube and connection are evacuated through stopcock 2 by an auxiliary pump. After stopcock 2 has been closed, the sample tube is filled with hydrogen-*t* from the manifold. The temperature and pressure are then measured. Next, 1 is closed (to trap a sample of the gas between 1 and 2). Residual gas in the line connecting the manifold is drawn into the Toepler pump, and the stopcock of the manifold is closed. Finally, the sample tube is removed from the manifold.

For radioassay, this gas is transferred to the ionization chamber with the apparatus of figure 3(b) in the following manner. The ionization chamber, *A*, is evacuated through stopcock 2. Then 2 is turned to allow gas from the sample tube to flow into the chamber. The line from a cylinder of hydrogen to 1 is first evacuated and then filled with hydrogen. Hydrogen is allowed to pass slowly, through 1 and the sample tube, into the ionization chamber, until atmospheric pressure is reached; then the stopcock on the ionization chamber is closed. The ion current arising from the sample in the ionization chamber is measured with a vibrating-reed electrometer in the customary manner [21]. The amount of tritium in the sample is calculated with a factor that is obtained by assaying hydrogen-*t* prepared from the NBS tritium oxide standard or from any source of known tritium content.

### 2.4. Conversion of Hydrogen-*t* to Water-*t*

To convert hydrogen-*t* to water-*t*, the copper oxide furnace (*F–G*) of figure 1 is heated to 500° C. The stopcocks are set so that hydrogen-*t* can pass from the gas buret *B* through the furnace and the water trap (*I*–*J*) (cooled by a dry-ice bath). The Toepler pump is started and the gas is circulated until the pressure in the system becomes constant. If desired, the water-*t* that collects in the trap can be diluted with water distilled from flask *E.* Finally, the trap containing the water-*t* is isolated by closing stopcocks *i* and *j* and opening stopcock 4. The water-*t* is stored in the trap until it is used. Any residual hydrogen-*t* in the system is diluted with hydrogen from *B.* The mixture is then oxidized by passing it through the copper oxide furnace and a second water trap (*L*–*M*) (cooled by a dry-ice bath). The gas is again circulated by the Toepler pump until the pressure in the system becomes constant. Finally, stopcocks *l* and *m* are closed and the water-*t* of low activity is stored in the trap until needed.

**Figure 4:**
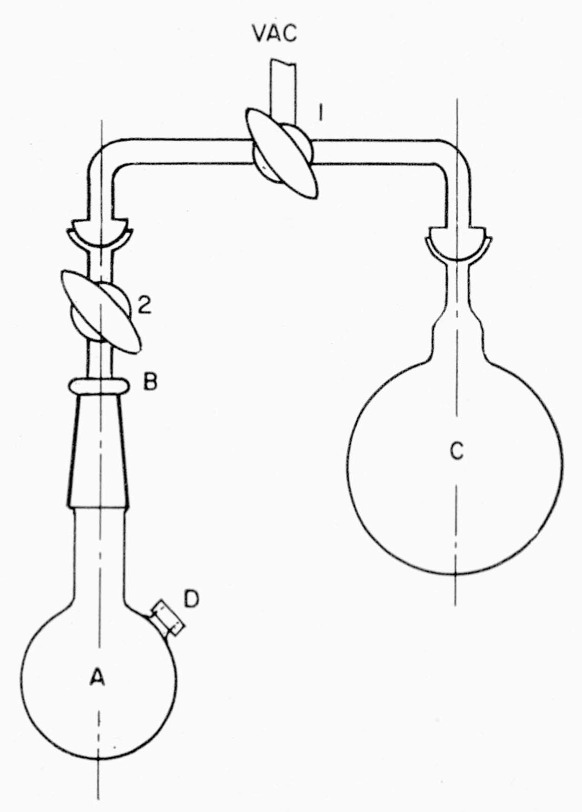
Adapter and flask for use with injection technique.

Water-*t* can be distilled from the trap into a receiver attached to the manifold at *k.* Water of high activity is collected and sealed in a break-seal receiving tube; it must be used in a closed system. Water of low activity is collected in a receiving flask such as *A* of figure 4. The receiving flask has a side arm closed with a rubber cap suitable for the addition or withdrawal of liquid by means of a hypodermic syringe. Under a suitable hood, samples of low-activity water can be withdrawn from the flask by a micropipet of the syringe type, and used as desired.

### 2.5. Assay of Water-*t* With a Proportional Counter

A definite quantity of water-*t*, less than 0.5 ml containing at least 10 *μ*c of tritium, is placed in a 5-ml volumetric flask. The water-*t* is frozen, and the flask is partially filled with a solution prepared from 175 g of sulfuric acid and 25 g of phosphoric anhydride. The mixture is warmed to room temperature; the volume is adjusted to 5 ml with the sulfuric acid solvent, and the solution is mixed. Then approximately 1 ml of the solution is transferred to a stainless-steel cell and counted in a a 2*π*, windowless, gas-flow, proportional counter. With the cell and equipment used for assay of carbon-14 [23], the counting rate was found to be 5.75 cps per *μ*c of tritium per ml of solution. This corresponds to a counting efficiency of only about 0.0155 percent. The low efficiency limits the method to assay of relatively high-activity materials.

The phosphoric anhydride-sulfuric acid solvent can be used for assay of a variety of tritium compounds. To avoid absorption of moisture, the solution must be transferred to the counting cell quickly, and the counting gas must be dried with phosphoric anhydride.

### 2.6. Assay of Water-*t* as Hydrogen-*t* in an Ionization Chamber

**Figure 5:**
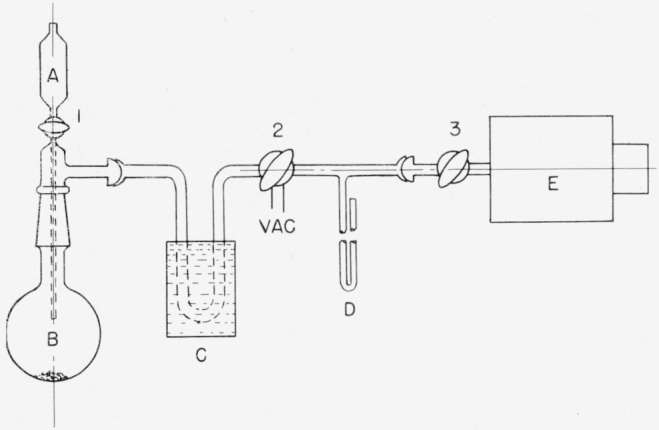
Apparatus for preparation and assay of hydrogen-t *A* Dropping funnel containing sample of water-*t* in 5-percent aqueous hydrochloric acid. *B* Flask containing 0.1 g of magnesium turnings. *C* Water trap cooled by dry-ice—ethyl alcohol bath. *D* Manometer. *E* Ionization chamber.

Methods used for gas counting of tritium prior to 1958 are reviewed in [19]. In the present study, satisfactory results have been obtained with the apparatus of figure 5 and the procedure described below. This consists of diluting water-*t* with aqueous acid, and generating hydrogen-*t* by allowing the acid to react with metallic magnesium.

A definite volume, or weight, of water-*t*, containing about 3 *μ*c of tritium, is diluted to 10 ml with 5-percent aqueous sulfuric acid and the solution is placed in flask *A* of figure 5. About 1 g of magnesium turnings is placed in flask *B.* Then the ionization chamber *E* and auxiliary apparatus are evacuated through stopcock 2 to a pressure of less than 0.1 mm of mercury. After the vacuum line has been closed, the tritiated aqueous acid is slowly added to *B*, until sufficient hydrogen-*t* has been generated to bring the system to atmospheric pressure. The pressure and temperature are read, and stopcock 3 is closed. Then the ionization chamber is connected to a vibrating-reed electrometer and the drift rate is measured in the customary manner. The radioactivity of the sample is evaluated by comparison of the drift rate with that in a control experiment run with a water sample of known tritium content (e.g., NBS Standard Sample 4926). The results are adjusted to correct for variation of temperature and pressure from standard conditions.

### 2.7. Apparatus for Dissolving, Filtering, and Freezedrying Materials in a Closed System^7^

**Figure 6:**
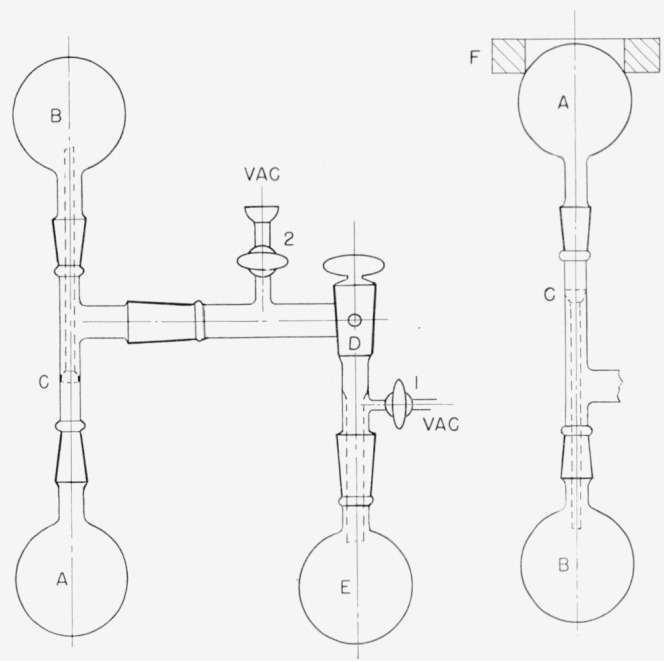
Apparatus for preparing solutions of materials in a closed system. *A* and *B* $\bar{\$}$ Flasks. *C* Filter constructed from funnel and a 20-mm, sintered-glass filter disk. *D* 10-mm Right-angle stopcock. *E* 200-ml $\bar{\$}$ Flask. *F* Cork ring to hold dry ice in place. 1 and 2 Precision-grade stopcocks.

The apparatus of figure 6 can be used for a variety of operations. Flasks *A* and *B* are connected by a tube having a sealed-in, fritted-glass filter, *C*, and are joined to flask *E* through a 24/40 $\bar{\$}$ joint and a large-bore stopcock, *D.* In most applications, the material to be purified is placed in *A*, and the solvent in *E.* Flask *E* is cooled in liquid nitrogen and the system is evacuated through stopcock 1. After stopcock 1 is closed, sufficient solvent is distilled from *E* into *A* to dissolve the material. Stopcock *D* is closed, and the unit *A–B* is turned so that *A* is above *B.* Then *B* is cooled, and the solution passes from *A* through the filter to *B.* The residue on the filter is washed by placing a cork ring and a small quantity of powdered dry ice on the top of *A*; solvent condenses in *A* and ultimately drains back into *B.* After extraction of the material on the filter by this means, the solvent in *B* is transferred to *E* by distilling or freeze-drying. (For details, see the next section.) Such volatile solvents as ether, dioxane, liquid ammonia, liquid sulfur dioxide, and water may be used. Nonvolatile reagents may be placed in either *A* or *B*, and solvents and reagents may be distilled into either *A* or *B* from *E* or from sources attached at stopcock 2. Solutions may be stirred with a magnetic stirring bar, placed in either *A or B* before evacuation. The device may be attached to the general-purpose manifold at 2, and volatile substances may be introduced or removed at will.

### 2.8. Preparation of Lithium Borohydride-*t*

As shown by Brown, Kaplan, and Wilzbach [28], when lithium borohydride is heated with tritium gas at about 200° C, tritium-hydrogen exchange takes place and tritium-labeled lithium borohydride is formed. For efficient reaction, the lithium borohydride must be in a finely divided condition. Commercial lithium borohydride can be converted to a suitable powder with the apparatus of figure 6. To obtain dry ether about 0.2 g of lithium aluminum hydride and 100 ml of reagent-grade ether are placed in flask *E* of figure 6. When the evolution of hydrogen has subsided, 2 g of commercial lithium borohydride is transferred to *A* (in a dry box tilled with nitrogen). Then the apparatus of figure 6 is assembled and is connected to a vacuum pump through stopcock 1 and a trap cooled by liquid nitrogen. The ether in *E* is frozen with liquid nitrogen, and the system is evacuated. The vacuum line is closed off, and the liquid-nitrogen bath is moved from *E* to *A.* The ether from *E* distills into *A.* When most of the ether has been transferred, stopcock *D* is closed and *A* is brought to room temperature. The lithium borohydride dissolves; the resulting solution is filtered to remove insoluble matter by turning the unit, carrying *A* and *B*, through 180° about the horizontal axis and then cooling *B.* This causes the liquid to drain from *A* into *B.* When the solution has collected in *B*, a cork ring and a few grams of dry ice are placed on *A.* Then ether distills from *B* into *A* and collects above the fritted-glass filter. When solvent sufficient for washing the residue on the filter has condensed, the cork ring and dry ice are removed and flask *B* is cooled. The solvent drains into *B*, washing the residue on the filter. Finally, the solvent is removed from *B* by opening stopcock *D*, cooling *E* with liquid nitrogen, and warming *B.* Care must be used to avoid bumping. When all of the solvent has been transferred to *E*, the last trace of solvent is removed through the vacuum line by heating *B* (in a silicone oil bath) to 200° C. Finally, stopcock *D* is closed and dry nitrogen is introduced through stopcock 2. The resulting product is a friable powder suitable for “labeling.”

**Figure 7:**
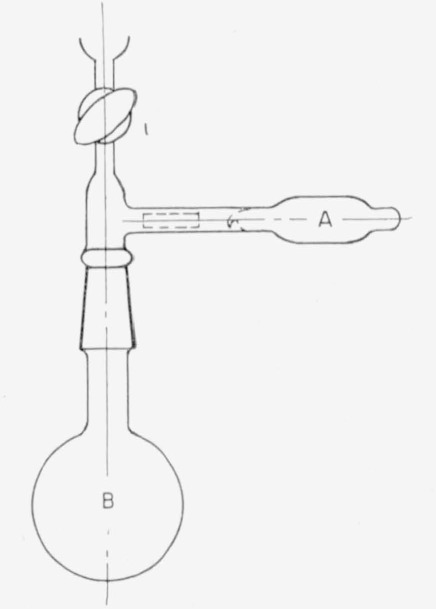
Apparatus for treating solids with tritium gas.

To make tritium-labeled lithium borohydride by tritium exchange [28], flask *B*, containing finely divided lithium borohydride, is connected to a source of tritium as shown in figure 7. The apparatus is attached to the general-purpose manifold (fig. 1 at *c*), made vacuum tight, and evacuated. Stopcock 1 and the stopcock to the manifold are closed, the break-seal to the tritium is broken, and flask *B* containing the lithium borohydride is heated in a silicone oil bath at 200° C. After 96 hr, the flask is opened to the manifold, and the hydrogen-tritium mixture in *B* is transferred by the Toepler pump to a receiver attached to the manifold. All traces of tritium are removed by introducing hydrogen from the manifold and re-evacuating. Finally, dry nitrogen is introduced into flask *B* from a source connected to the manifold. Then flask *B* is removed, stoppered, and kept in a dry box until the labeled lithium borohydride is to be used. The specific activity of the product depends on the relative proportions of tritium and lithium borohydride used. A preparation derived from 2 g of lithium borohydride and 4 curies of tritium gave a product having an activity of 2.94 curies. Thus, 73 percent of the tritium used was fixed in the hydride.

### 2.9. Preparation and Use of Solutions of Lithium Borohydride-*t*

Under nitrogen in a dry box, the desired amount of lithium borohydride-*t* (from flask *B*, described in the preceding section) is placed in flask *A* of figure 4. The flask is closed (in the dry box) with the adapter, *B.* The side arm of *A* is closed with a rubber stopper, *D*, suitable for perforation with a hypodermic needle. The flask and adapter are weighed before and after introduction of the lithium borohydride. The flask is then connected, as shown in figure 4, to flask *C*, containing tetrahydrofuran and sufficient lithium aluminum hydride to remove all moisture and other reactive contaminants. About 20 ml of tetrahydrofuran is used for each 100 mg of lithium borohydride in flask *A.* The tetrahydrofuran in *C* is frozen in liquid nitrogen, and the system (including *A*) is evacuated. Then the tetrahydrofuran from *C* is distilled into *A*, by warming *C* and cooling *A.* After the solvent has been transferred to *A*, stopcock 2 is closed and *A* (including adapter *B*) is disconnected from *C.*

Reductions can be performed directly in flask *A*. A solution of the material to be reduced is added through the stopper *D* by means of a hypodermic syringe fitted with a stainless-steel needle. When reduction is complete, the reaction flask is connected to the manifold of figure 1 and any hydrogen-*t* present is transferred by the Toepler pump to a reservoir attached to the manifold. The nonvolatile product is isolated in a manner appropriate to the particular substance (see sec. 2.11). In the event that several reductions are to be performed, portions of the lithium borohydride solution in *A* are withdrawn with a graduated syringe and injected into several evacuated reaction flasks (like that shown by *A* of fig. 4) containing the material to be reduced.

### 2.10. Analysis of a Solution of Lithium Borohydride-*t*

**Figure 8:**
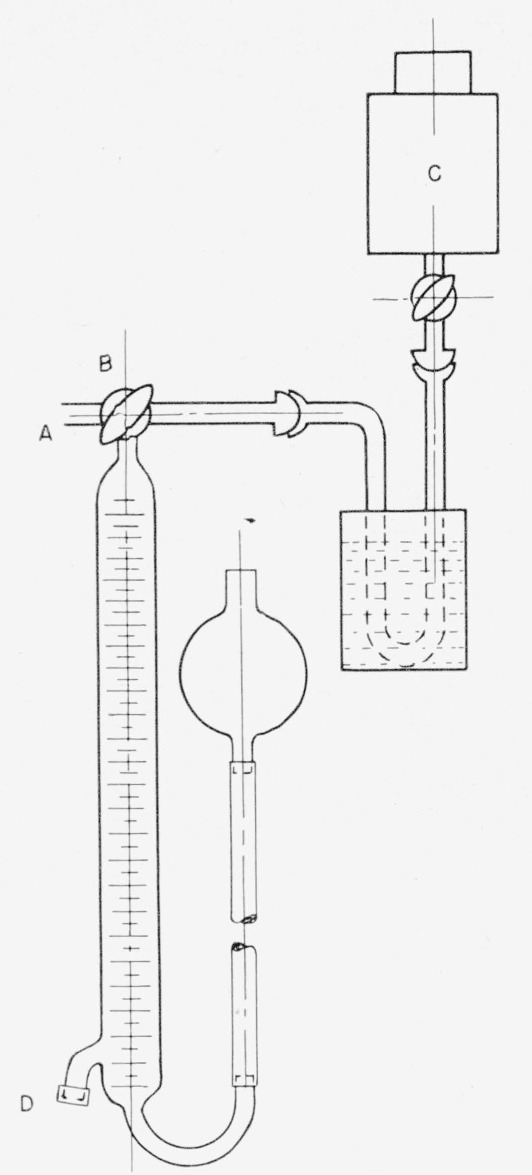
Apparatus for assay of metal hydrides.

For analysis, a known volume of the solution of lithium borohydride-*t* in tetrahydrofuran is injected into 5-percent aqueous hydrochloric acid contained in the gas buret of figure 8. The injection is made through a rubber cap, *D*, near the bottom of the buret. A small amount of mercury is placed in *D*, to prevent leakage. The hydrogen-*t* is collected in the gas buret, and the radioactivity is measured by transferring the gas from the gas buret to an evacuated ionization chamber, with the arrangements shown in figure 8. After introduction of the sample, the buret is flushed with hydrogen generated by injection of a solution of nonradioactive sodium borohydride at *D.* Finally, the ionization chamber, *C*, is filled to atmospheric pressure with hydrogen, and the radioactivity is measured with an electrometer in the conventional manner.

The specific activity of lithium borohydride-*t* can be estimated from the specific activity of a product made from it, as, for example, d-galactitol-*1-t* described in the next section. This method of analysis, although useful, is subject to error from isotope effects.

### 2.11. Preparation of d-Galactitol-*1*-*t*

A magnetic stirring bar, two millimoles of d-galactose, and one millimole of sodium carbonate are placed in a 50-ml flask of the type shown in figure 4. The flask is connected to the general-purpose manifold (fig. 1) and evacuated. Then the connection to the manifold is closed; the flask is cooled by an ice bath, placed on the driving mechanism of the stirrer. The stirrer is started, and 2 ml of ice water is added by injection with a hypodermic needle, through the rubber stopper of the flask. This is followed by addition, in the same manner, of 2 ml of a solution containing 12 mg of lithium borohydride-*t* (1.47 mc/mg) in dry tetrahydrofuran. Stirring is continued for about 1 hr at ice temperature, and then the solution is allowed to warm to room temperature. After 18 hr, 1 ml of 5-percent aqueous acetic acid is added to decompose any unreacted lithium borohydride; the solution is frozen in a liquid-nitrogen bath, and any hydrogen-*t* present is collected in the manifold. Finally, the connection to the manifold is closed and the flask is removed from the manifold. The solvent is removed by the freeze-drying technique, and, ultimately, transferred to a closed container used for disposal of radioactive waste. The residue containing the product is dissolved in 10 ml of water, and the solution is evaporated to dryness in a rotary still. The residue is dissolved in water and the solution is passed through a column containing 10 ml of a cation-exchange resin.^8^ The effluent is evaporated to dryness on a vacuum rotary still; the residue is dissolved in 15 ml of methanol and the solvent is evaporated. Dissolution in methanol and evaporation are repeated 4 times to remove all of the boric acid as methyl borate. The residue consists of d-galactitol-*1*-*t* and a small quantity of unreacted d-galactose. It is recrystallized from 15 ml of hot ethanol. In a preparation conducted as described, the crude product contained 16 mc of tritium, and the yield of recrystallized d-galactitol-*1-t* was about 90 percent of the theroretical. The d-galactitol-*1-t* was assayed for radioactivity by the method of Isbell, Frush and Peterson [17]. A 0.00025-mg sample in a sodium *O*-(carboxymethyl) cellulose film weighing 19.8 mg gave 126 counts per second. This corresponds to an activity of 44 *μ*c/mg.

## Footnotes

2 Figures in brackets indicate the literature references at the end of this paper.

3 Published research on deuterium and tritium compounds prior to 1956 is listed in a series of annual bibliographies prepared by the Thermodynamics Section of NBS [22] under the auspices of the Atomic Energy Commission.

4 The product is not a *meso* compound, but a true derivative of d-galactitol.

5 The manifold and auxiliary equipment are more or less conventional, and ideas for their construction were drawn from many sources. Textbooks by Glascock [24] and by Sanderson [25] were particularly helpful.

6 Stopcocks must be of high quality and carefully lubricated with vacuum grease. Ball-joints are sealed with vacuum-grade sealing wax.

7 An excellent review by R. S. Tipson [27] describes a variety of schemes for effecting crystallization and filtration in closed systems. Some of these are similar to. but not the same as, that described here.

8 Amberlite IR 120-H, Rohm and Haas Co., Philadelphia, Pa.
